# *LAZY* Gene Family in Plant Gravitropism

**DOI:** 10.3389/fpls.2020.606241

**Published:** 2021-01-15

**Authors:** Zhicheng Jiao, Huan Du, Shu Chen, Wei Huang, Liangfa Ge

**Affiliations:** ^1^College of Forestry and Landscape Architecture, South China Agricultural University, Guangzhou, China; ^2^Guangdong Key Laboratory for Innovative Development and Utilization of Forest Plant Germplasm, South China Agricultural University, Guangzhou, China; ^3^State Key Laboratory for Conservation and Utilization of Subtropical Agro-bioresources, College of Life Sciences, South China Agricultural University, Guangzhou, China; ^4^Guangdong Provincial Key Laboratory of Protein Function and Regulation in Agricultural Organisms, College of Life Sciences, South China Agricultural University, Guangzhou, China; ^5^The Guangdong Subcenter of the National Center for Soybean Improvement, College of Agriculture, South China Agricultural University, Guangzhou, China

**Keywords:** gravitropism, *LAZY*, auxin, PIN, gene family

## Abstract

Adapting to the omnipresent gravitational field was a fundamental basis driving the flourishing of terrestrial plants on the Earth. Plants have evolved a remarkable capability that not only allows them to live and develop within the Earth’s gravity field, but it also enables them to use the gravity vector to guide the growth of roots and shoots, in a process known as gravitropism. Triggered by gravistimulation, plant gravitropism is a highly complex, multistep process that requires many organelles and players to function in an intricate coordinated way. Although this process has been studied for several 100 years, much remains unclear, particularly the early events that trigger the relocation of the auxin efflux carrier PIN-FORMED (PIN) proteins, which presumably leads to the asymmetrical redistribution of auxin. In the past decade, the *LAZY* gene family has been identified as a crucial player that ensures the proper redistribution of auxin and a normal tropic response for both roots and shoots upon gravistimulation. LAZY proteins appear to be participating in the early steps of gravity signaling, as the mutation of *LAZY* genes consistently leads to altered auxin redistribution in multiple plant species. The identification and characterization of the *LAZY* gene family have significantly advanced our understanding of plant gravitropism, and opened new frontiers of investigation into the novel molecular details of the early events of gravitropism. Here we review current knowledge of the *LAZY* gene family and the mechanism modulated by LAZY proteins for controlling both roots and shoots gravitropism. We also discuss the evolutionary significance and conservation of the *LAZY* gene family in plants.

## Plant Gravitropism

**FIGURE 1:**
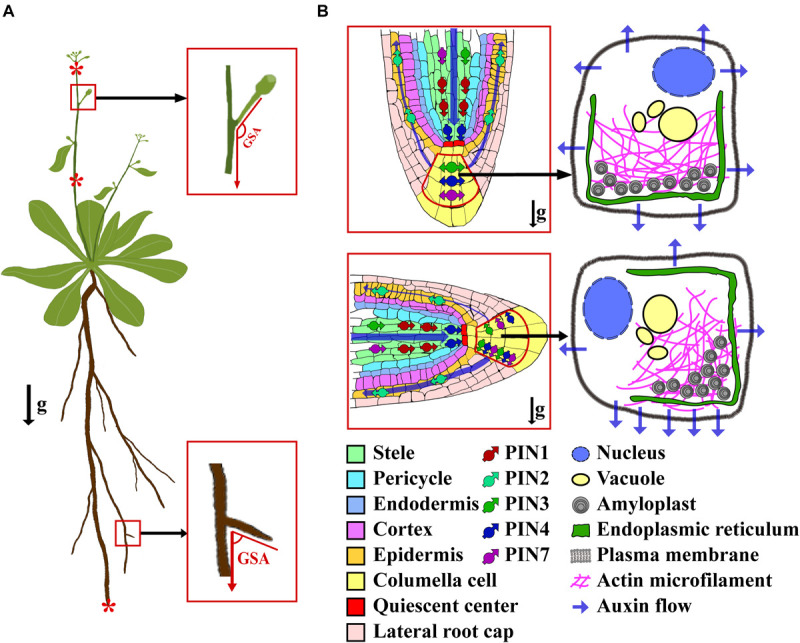
The diagram of GSA and gravitropism of root tip. **(A)** A plant showing the concept of GSA and gravity-sensing tissues in shoot and root. The asterisks (from top to bottom) show the representative gravity-sensing tissues of inflorescence, shoot, and root, respectively. **(B)** A schematic showing the activity of PINs and auxin flow in a root tip before (top) and after (bottom) a 90° reorientation. After a gravistimulation by the 90° reorientation, the PINs within the columella cell are polarized along with the direction of the gravity, and auxin is asymmetrically transported to the bottom side of the root tip. The arrows of g indicate the direction of gravity vector.

The growth angle of plant organs is a fundamental component of plants’ shoot and root system architecture, and it largely defines the space that a plant can access. The angle of a plant organ is influenced by both its intrinsic genetic nature and a variety of environmental factors, including gravitation pull, light direction, temperature, and wind. Within the gravity field, plants can sense gravitational pull and adjust their organs’ growth direction accordingly. Consequently, plant organs are developed and maintained at particular angles, which are called the gravitropic set-point angles (GSAs) (Digby and Firn, 1995; Figure 1A).

Plant gravitropism can either be a positive or a negative response (Dong et al., 2013). The positive gravitropic response occurs in the belowground root system, which re-orientates the root-tip growth angle toward the GSA and allows the roots to penetrate deep into the soil to anchor the plant and uptake nutrients and water. By contrast, the negative gravitropic response occurs in the aboveground shoot system, which grows up, against the gravity vector toward the sky to obtain space and sunlight for photosynthesis and reproduction.

Plant gravitropism comprises four successive steps: gravity perception, signal transduction that converts the gravity stimulus into physiological signals, polar auxin transport (PAT) that asymmetrically distributes auxin to the responding organs, and differential elongation of cells leading to the curvature of organs. How exactly the plant perceives the gravitational pull is not yet fully understood. The starch-statolith theory posits that gravity is perceived by the statoliths within the statocyte cells. These statocytes contain amyloplasts, which are filled with dense starch, thereby conferring to them a greater density than the surrounding cytoplasm (Baldwin et al., 2013; Su et al., 2017). Upon encountering gravitational pull, the amyloplasts can rapidly descend toward the new bottom of the cell, triggering the downstream gravitropism signal transduction via an as of yet unclear mechanism. Notably, the sedimentation process of the amyloplasts is finely tuned by the actin microfilaments network (Hou et al., 2003; Leitz et al., 2009; Blancaflor, 2013). In roots, the amyloplasts’ sedimentation occurs in the columella cells. Following downward migration and settling of amyloplasts in the columella cells, this physical stimulus is transduced into a physiological and biochemical signal (Figure 1B). Unfortunately, the nature and mechanism of that signal remain unresolved. It has been proposed that the signal is capable of triggering the lateral relocation of LAZY proteins and auxin transporters (Friml et al., 2002; Furutani et al., 2020), which further asymmetrically transport auxin to the elongation zone, where the root tip bends toward the gravity vector (Blancaflor and Masson, 2003; Morita and Tasaka, 2004; Blancaflor, 2013). In shoots, negative gravitropism takes place in several tissues, including inflorescence stems, epicotyls, and hypocotyls in dicots, and the culms, mesocotyl-coleoptiles, and pulvini in monocots (Imagawa et al., 1991; Blancaflor and Masson, 2003; Morita and Tasaka, 2004; Haga and Iino, 2006; Li et al., 2007; Blancaflor, 2013; Dong et al., 2013). In these tissues, the amyloplasts’ sedimentation has been directly observed in certain types of cells (such as the endodermis) (Morita and Tasaka, 2004; Saito et al., 2005; Swarup et al., 2005; Su et al., 2017). The sediment of amyloplasts in shoot tissues then triggers the lateral transport of auxin across the organ by an unclear mechanism, which leads to the asymmetrical accumulation of auxin in the opposite flanks of the responding organ, and ultimately curvature of the organ toward the GSA within hours (Su et al., 2017).

## Gravitropism Regulates Plant Architecture and Performance

**FIGURE 2:**
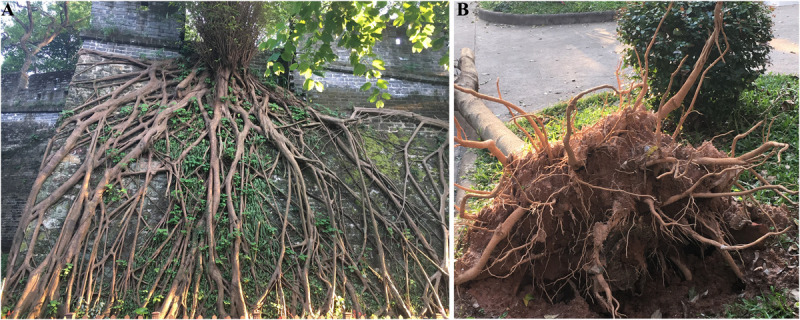
Deep roots and shallow roots. **(A)** A tree with deep roots on the ancient city wall in Guangzhou Yuexiu Park. **(B)** A tree with shallow roots that was toppled by the strong winds of “Typhoon Mangkhut.”

Gravitropism has a profound influence on both the shoot and root systems’ architecture. As the most stable environmental signal, the gravity vector continuously guides the growth direction of all plant organs, thus shaping not only the overall form of a given plant but also the various characteristics of its organs, such as their growth angle, orientation, and pattern. Many studies have reported on how an altered responsiveness to the gravistimulation can lead to varied plant architectures. For example, a mutation of *Loose Plant Architecture1* (*LPA1*) affected the perception of gravistimulation, resulting in a loose shoot architecture forming in rice, in contrast to the rather compact shoot system of the wild type (Wu et al., 2013). In roots, the strength of their gravitropic response largely defines the root-tip growth angle, root depth, and distribution of roots in the soil. Disrupting or reducing gravitropism could lead to roots failing to respond properly to the perceived gravitational pull or a shallow root depth (Luschnig et al., 1998; Muller et al., 1998; Rouse et al., 1998; Kitomi et al., 2020). In nature, roots display highly varied depths (Figure 2), which convey divergent or disparate genetic abilities to perform gravitropism. Rooting depth also dramatically affects plant performance, particularly under certain circumstances. Shallow roots, which may have relatively low access to available water under drought conditions (Uga et al., 2013), as well as low wind resistance and lodging resistance (Figure 2), can nonetheless increase the acquisition of certain nutrients in topsoil layer (Wang et al., 2010; Lynch, 2011), and they may even increase yield in saline soils by modulating gravitropic responses when roots grow on the soil-surface (Kitomi et al., 2020). The architecture of both shoots and roots plays a determinant role in the performance of crops and other economic plants; hence, the importance of understanding gravitropism for their improvement.

## *Lazy* Genes Regulate Plant Gravitropism in Both Shoot and Root

Despite much progress having been made in understanding plant gravitropism, there are still many unsolved core questions in the current four-step gravitropism model, particularly the early events spanning from gravity perception to signal transduction (Su et al., 2017). For decades, the starch-statolith theory has been popular for explaining the initial process of gravitropism; there is evidence indicating it may not apply to all species (Edelmann, 2018; Richter et al., 2019; Ditengou et al., 2020). Thus, identifying new players involved in these early events would be vital to understanding the molecular architecture characterizing the early steps of plant gravitropism.

In the past decades, the *LAZY* gene family has been identified as the key regulator in both roots’ positive gravitropism and shoots’ negative gravitropism. *LAZY* genes have been designated with other names apart from *LAZY*, such as *NEGATIVE GRAVITROPIC RESPONSE OF ROOTS* (*NGR*), and *DEEPER ROOTING* (*DRO*). A previous review suggested using *LZY* as the representative nomenclature (Nakamura et al., 2019), so hereon we refer to *LZY* for consistency.

**FIGURE 3:**
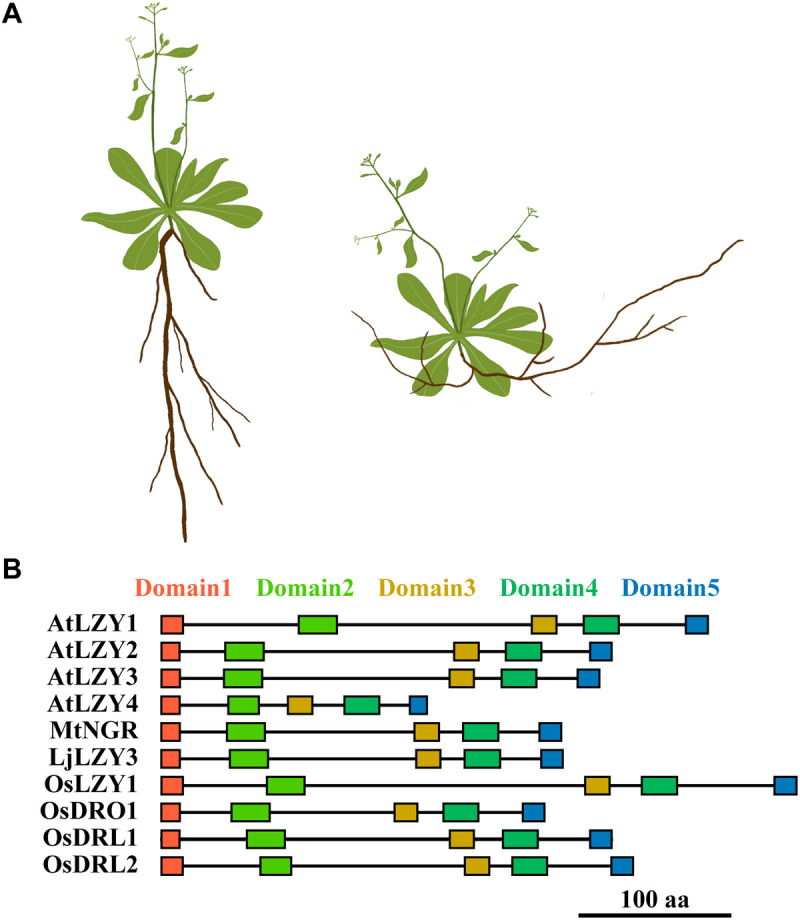
A diagram shows the phenotypic changes caused by the mutation of *LZY*s and the arrangement of the conserved domains of the representative LZY homologs. **(A)** The shoot and root phenotype of a wide type (left) and mutant (right) plant. The mutation of *LZY*s caused an impaired gravitropic response in shoot and a negative gravitropic response in roots, respectively. Consequently, the branches of the shoot declined toward the ground, and the roots gained negative gravitropism that leads to the roots growing toward the sky. **(B)** The rectangles indicate the five conserved domains of LZY proteins.

The name *lzy* originates from those classical rice and maize plants deemed “lazy,” which developed spreading tillers in rice or prostate culms in maize (Overbeek, 1936; Jones and Adair, 1938). Phenotypic characterization and physiological studies indicated that the negative gravitropic response of the aboveground shoot of these plants had been severely impaired (Li et al., 2007; Yoshihara and Iino, 2007; Dong et al., 2013). Upon gravitational pull, the horizontally placed hypocotyl, coleoptile, and inflorescence of the *lzy1* plants bent more slowly than did their counterparts in wild-type plants. In this way, the shoot grew horizontally or with a large spreading angle under gravitational pull (Overbeek, 1936; Jones and Adair, 1938; Li et al., 2007; Dong et al., 2013). The *lzy1* mutant was also identified in Arabidopsis and designated as *atlzy1*; its branching has outward orientation and wider angles than do the branches of the wild type (Yoshihara et al., 2013; Figure 3A). This growth habit resembles the rice *lzy1* mutant and was caused by impaired gravitropic responses, which also led to the slowed dynamics of gravitational curvature of the horizontally placed *atlzy1* plants.

Although root gravitropism functioned normally in the *lzy1* mutants of rice, maize, and Arabidopsis, a few recent reports have shown that members of the *LZY* gene family are also crucial regulators of the roots’ positive gravitropism in several species (Uga et al., 2013; Ge and Chen, 2016; Taniguchi et al., 2017; Yoshihara and Spalding, 2017; Ge and Chen, 2019; Chen et al., 2020; Figure 3A). Disruption of one of the *LZY* homologs in two legume species, *Medicago truncatula* and *Lotus japonicus* reversed the positive gravitropism of their roots to negative gravitropism, resulting in roots that grew upward and emerged above the soil surface (Ge and Chen, 2016; Chen et al., 2020). Additionally, simultaneous mutation of three *LZY* genes in Arabidopsis also led to upwardly growing roots, clearly a negative gravitropic response, thus resembling the mutants identified in *M. truncatula* and *L. japonicus* (Ge and Chen, 2016; Figure 3A). The rice genome carries four *LZY*s, namely, *LZY1*, *DRO1*, *DRO1-like 1* (*qSOR1*, *DRL1*), and *DRO1-like 2* (*DRL2*) (Ge and Chen, 2016; Kitomi et al., 2020; Table 1). Natural mutation of DRO1 and DRL1, and a knockout mutation of DRL2 by CRISPR/Cas9 technology all led to impaired root gravitropic responses and an altered RSA, even with soil-surface roots (SOR) (Kitomi et al., 2020).

**TABLE 1:** LZYs in Arabidopsis (*A. thaliana*), soybean (*G. max*), lotus (*L. japonicus*), Medicago (*M. truncatula*), and rice (*O. sativa*).

**Gene Name**	**Locus**	**Species**	**Similarest Arabidopsis Gene**	***E*-value**	**References**
*AtLZY1*	AT5G14090	*A. thaliana*			Yoshihara et al., 2013
*AtLZY2*	AT1G17400	*A. thaliana*			Yoshihara et al., 2013; Ge and Chen, 2016
*AtLZY3*	AT1G72490	*A. thaliana*			Yoshihara et al., 2013; Ge and Chen, 2016
*AtLZY4*	AT1G19115	*A. thaliana*			Yoshihara et al., 2013; Ge and Chen, 2016
*AtLZY5*	AT3G24750	*A. thaliana*			Yoshihara et al., 2013
*AtLZY6*	AT3G27025	*A. thaliana*			Yoshihara et al., 2013
*GmLZY1*	Glyma.02G097100	*G. max*	*AtLZY1*	1.43E-50	This study
*GmLZY2*	Glyma.10G298100	*G. max*	*AtLZY1*	9.34E-43	This study
*GmLZY3*	Glyma.16G054300	*G. max*	*AtLZY1*	1.99E-54	This study
*GmLZY4*	Glyma.18G284400	*G. max*	*AtLZY1*	3.37E-51	This study
*GmLZY5*	Glyma.19G094600	*G. max*	*AtLZY1*	5.22E-55	This study
*GmLZY6*	Glyma.20G249200	*G. max*	*AtLZY1*	3.5E-41	This study
*GmLZY7*	Glyma.03G264200	*G. max*	*AtLZY3*	2.95E-81	Ge and Chen, 2016
*GmLZY8*	Glyma.07G040800	*G. max*	*AtLZY3*	5.82E-96	Ge and Chen, 2016
*GmLZY9*	Glyma.09G071100	*G. max*	*AtLZY3*	9.96E-35	This study
*GmLZY10*	Glyma.15G179200	*G. max*	*AtLZY3*	1.72E-33	This study
*GmLZY11*	Glyma.16G009400	*G. max*	*AtLZY3*	1.59E-96	Ge and Chen, 2016
*GmLZY12*	Glyma.19G263200	*G. max*	*AtLZY3*	3.02E-86	Ge and Chen, 2016
*GmLZY13*	Glyma.08G356200	*G. max*	*AtLZY5*	5.12E-17	This study
*GmLZY14*	Glyma.16G094000	*G. max*	*AtLZY5*	7.59E-10	This study
*GmLZY15*	Glyma.18G173300	*G. max*	*AtLZY5*	5.73E-16	This study
*LjLZY1*	Lj1g3v3171170	*L. japonicus*	*AtLZY1*	2E-50	Chen et al., 2020
*LjLZY2*	Lj1g3v3466030	*L. japonicus*	*AtLZY5*	2E-16	Chen et al., 2020
*LjLZY3*	Lj3g3v2576600	*L. japonicus*	*AtLZY3*	9E-79	Ge and Chen, 2016; Chen et al., 2020
*LjLZY4*	Lj6g3v1177230	*L. japonicus*	*AtLZY2*	2E-28	Chen et al., 2020
*LjLZY5*	Lj0g3v0142099	*L. japonicus*	*AtLZY5*	2E-10	Chen et al., 2020
*LjLZY6*	Lj0g3v0338439	*L. japonicus*	*AtLZY1*	2E-25	Chen et al., 2020
*MtLZY7*	MtrunA17_Chr2g0301041	*M. truncatula*	*AtLZY2*	4.27E-09	This study
*MtLZY3*	MtrunA17_Chr3g0122881	*M. truncatula*	*AtLZY1*	1.91E-43	This study
*MtLZY2*	MtrunA17_Chr7g0222391	*M. truncatula*	*AtLZY1*	1.24E-45	This study
*MtLZY4*	MtrunA17_Chr7g0224671	*M. truncatula*	*AtLZY1*	1.31E-26	This study
*MtLZY1*	MtrunA17_Chr7g0224731	*M. truncatula*	*AtLZY1*	3.4E-56	This study
*MtLZY6*	MtrunA17_Chr7g0237671	*M. truncatula*	*AtLZY5*	8.2E-19	This study
*MtLZY5*	MtrunA17_Chr7g0240781	*M. truncatula*	*AtLZY5*	9.09E-20	This study
*MtNGR*	MtrunA17_Chr8g0344431	*M. truncatula*	*AtLZY3*	4.7E-98	Ge and Chen, 2016
*OsDRO1*	LOC_Os09g26840	*O. sativa*	*AtLZY3*	6.11E-35	Uga et al., 2013; Ge and Chen, 2016
*OsLZY1*	LOC_Os11g29840	*O. sativa*	*AtLZY1*	2.48E-15	Li et al., 2007
*OsDRL2*	LOC_Os03g29270	*O. sativa*	*AtLZY3*	3.43E-29	Ge and Chen, 2016; Kitomi et al., 2020
*OsDRL1*	LOC_Os07g42290	*O. sativa*	*AtLZY3*	4.99E-39	Ge and Chen, 2016; Kitomi et al., 2020

Altogether, these findings strongly demonstrated the key involvement of *LZYs* in both shoot and root gravitropism. Notably, though functions can be altered by the mutation of *LZYs*, their outcomes differ in plants. In the aboveground shoots, mutation of *LZY1* impairs or weakens shoots’ negative gravitropic responses, whereas in roots, the positive gravitropism was reversed into negative gravitropism via mutation of *LZY* homologs.

## LZY Proteins Regulate Pat and Asymmetric Auxin Distribution

Intriguingly, the sedimentation of amyloplasts in the above-reported *lzy* mutants was all normal (Abe et al., 1994; Li et al., 2007; Ge and Chen, 2016; Taniguchi et al., 2017), whereas PAT was dramatically altered in them. In both Arabidopsis *atlzy2,3,4* and *L. japonicus ljlzy3* mutants, whose roots grew upward, the asymmetrical auxin distribution in the elongation zone of these mutated roots has been completely reversed, leading to more auxin accumulation in the new top flank upon gravistimulation; this contrasts starkly with the wild-type roots, in which the higher accumulation of auxin was found in the new bottom flank (Ge and Chen, 2016; Taniguchi et al., 2017; Yoshihara and Spalding, 2017; Chen et al., 2020). Also, the auxin efflux carrier PIN-FORMED 3 (PIN3) which is located in the columella cells and is capable of polarizing to redirect auxin flux upon gravistimulation, was reversely polarized in the Arabidopsis *atlzy2,3,4* mutant. Moreover, accumulation of the PIN2 protein, which is located in the epidermal and cortex cells of the root tip and crucial for asymmetric auxin flow of root gravitropism was found predominantly at the top flank of *ljlzy3* mutant, the opposite of that in the wild-type roots (Chen et al., 2020).

Likewise, the asymmetrical auxin distribution was also severely impaired in the coleoptiles of rice *lzy1* and abolished in the coleoptiles of maize *la1-ref* mutant after incurring gravistimulation (Yoshihara and Iino, 2007; Dong et al., 2013). Additionally, the basipetal auxin transport in the coleoptiles of both rice *lzy1* and maize *la1-ref* mutant was strongly enhanced (Li et al., 2007; Yoshihara and Iino, 2007; Dong et al., 2013), indicating that PAT was altered somehow in *lzy1* mutants. Taken together, these findings from both shoots and roots consistently demonstrate that LZYs do not interfere with amyloplasts sedimentation, but rather play a key role in directing asymmetrical auxin distribution upon gravistimulation.

Asymmetrical auxin distribution relies on the polarization of auxin transporters, which is presumably able to trigger an asymmetrical auxin flux, thereby leading to further asymmetry in auxin accumulation (Dhonukshe et al., 2010). Thus, upon gravistimulation, transcytosis-based polarization of the auxin efflux carriers PIN3 and PIN7 in columella cells is essential for roots’ positive gravitropism (Dhonukshe et al., 2010). In Arabidopsis, mutation of *LZY*s reversed the polarization of PIN3 onto the new upper side of columella cells (Taniguchi et al., 2017; Ge and Chen, 2019). Moreover, disruption of PINs expressed in columella cells impaired the negative gravitropism of these mutants (Ge and Chen, 2019). These observations align well with the reversed asymmetry of auxin distribution and the negative gravitropic response of mutant roots, which indicates that LZYs regulate auxin distribution by regulating PIN polarization. Thus, LZYs can be defined as being important PINs regulators, directly or indirectly, which function to ensure PINs translocate to the correct side upon gravistimulation (Li et al., 2007).

## Conserved Domains in LZY Proteins

Although the overall similarity of LZY homologs is relatively low, LZYs proteins do share conserved molecular function. For example, the shared sequence identity between AtLZY1 and AtLZY2 is only 19.8%, yet AtLZY2 was still able to rescue the phenotype of *atlzy1* when driven by the promoter of *AtLZY1* (Yoshihara and Spalding, 2020). Perhaps this is possible because the LZYs share five conserved domains (Dardick et al., 2013; Yoshihara et al., 2013; Figure 3B), which seem to be decisive for the proper functioning of LZY proteins.

What are the exact functions of these five conserved domains? A recent study has thoroughly investigated the functions of the domains of AtLZY1 by mutating the amino acids. The domain I, which is located at the far N terminus of the protein and necessary for the plasma membrane localization of AtLZY1, appears to be vital for its function in regulating shoot branch angles. The domain II, harboring the highly conserved signature sequence of the family (Dardick et al., 2013; Yoshihara and Spalding, 2020), is crucial to the protein’s function. Once the conserved residues were mutated in Arabidopsis, the auxin gradient was reversed, such that the plant inflorescence gained positive gravitropism, showing the weeping shoot phenotype (Yoshihara and Spalding, 2020). The functions of domain III and IV are still unclear, as their mutation did not change either the subcellular localization or the function of AtLZY1 (Yoshihara and Spalding, 2020).

Domain V, located at the far C terminus of the proteins, contains an EAR-like motif (Dardick et al., 2013; Yoshihara et al., 2013; Ashraf et al., 2019), and has been termed a conserved C terminus in LZY (CCL) family proteins (Taniguchi et al., 2017) or a WIKTD motif (Dardick et al., 2013). This domain V is critical for the biological functioning of the protein. When mutated, the normal function of the protein was severely impaired, resulting in the failed rescue of the *atlzy1*, though the subcellular localization of the protein was unaffected (Yoshihara and Spalding, 2020). According to two recent reports, domain V can interact with the BREVIS RADIX (BRX) domain, which exists in the plant-specific BRX gene family and PRAF (PH, RCC1, and FYVE)-like family proteins, to mediate homotypic and heterotypic protein interactions (Briggs et al., 2006; Li et al., 2019; Furutani et al., 2020).

The presence of the EAR-like motif in domain V is rather interesting. The EAR motif can recruit the transcription suppressor TOPLESS (TPL) to suppress the expression of downstream genes via epigenetic regulation, and it is usually found among the transcription factors. A recent report has shown that this domain from wheat LZY homologs can interact with TPL in yeast two-hybrid and bimolecular fluorescence complementation (BiFC) assays (Ashraf et al., 2019), raising the possibility that the mechanic action of LZY family proteins might have a connection with TPL gene family and involves transcriptional regulation.

Overall, a few reports have revealed the role of domain V from various aspects, which undoubtedly supports its critical contribution to determining the function of LZY proteins, from their subcellular localization to the interaction with partner proteins, and even potential transcriptional regulation. Further analysis of the functioning of domain V *in planta* would help to answer questions of how exactly it functions and whether it is indeed actively involved in transcriptional regulation.

## Subcellular Localization of LZY Proteins

Subcellular localization of a protein is an essential piece of information to understand its molecular mechanism. Despite many efforts, the subcellular localization of LZY proteins is still not fully known. The major problem lies in their controversial localization pattern, i.e., plasma membrane versus nucleus. Protein subcellular prediction has found both transmembrane domain and nuclear localization signal (NLS) peptides in OsLZY1 and AtLZY1 (Li et al., 2007; Yoshihara et al., 2013). Whereas a transmembrane domain is a symbol of membrane protein, the NLS and EAR motif are typically found in transcription factors. Transient expression of LZYs fused with the green fluorescence protein (GFP) demonstrated that OsLZY1 and AtLZY1 are localized to both the plasma membrane and nucleus (Li et al., 2007; Yoshihara et al., 2013). Inducible expression of GFP-fused AtLZY1 in transgenic Arabidopsis also showed that AtLZY1 was localized to both the plasma membrane and nucleus (Li et al., 2007). Additionally, disruption of the NLS eliminated the nuclear localization (Li et al., 2007; Yoshihara et al., 2013), and deletion of the transmembrane domain removed the plasma membrane localization (Li et al., 2007). These findings collectively provide convincing evidences that OsLZY1 and AtLZY1 are localized to the plasma membrane and nucleus, though the underlying mechanism likely differs between them. The nucleus localization of AtLZY1 seemed inconsequential (Yoshihara et al., 2013), whereas that of OsLZY1 was clearly essential to its functioning (Li et al., 2019).

OsLZY1 interacts with a member of the rice Brevis Radix family, OsBRX Like 4 (OsBRXL4), on the plasma membrane. This interaction between OsLZY1 and OsBRXL4 was capable of affecting the nucleus localization of OsLZY1. Once the abundance of OsBRXL4 was increased by overexpressing, more OsLZY1 appeared on the plasma membrane with comparatively less found in the nucleus, and the transgenic plant displayed a broader tiller angle (Li et al., 2019). While proteins that localize to both plasma membrane and nucleus are not very common, there is a group of transcription factors, called membrane-bound transcription factors (MTFs), which sense the signal on the membrane and move into the nucleus to exert their regulatory function (Chen et al., 2008; Li et al., 2016). The dynamic partitioning of OsLZY1 protein between the plasma membrane and nucleus upon gravistimulation represents a new segment of the model addressing the role of OsLZY1 in regulating the tiller angles and shoot gravitropism in rice.

The subcellular localization of many other LZY homologs has also been investigated. These homologs, including AtLZY2, AtLZY3, AtLZY4, and OsDRL1, were mainly localized to the plasma membrane in transient expression assays using *Nicotiana benthamiana* leaf epidermal cells or protoplast cells (Taniguchi et al., 2017; Ge and Chen, 2019; Chen et al., 2020; Kitomi et al., 2020; Yoshihara and Spalding, 2020). The plasma membrane localization of LZYs lends support to the view that LZYs can regulate the polarization of PINs. However, these findings of subcellular localization were acquired from either transient expression or a snapshot of GFP-fused LZYs. Rather, direct observation of LZYs’ subcellular localization in statocyte cells is essential for understanding its mechanism (Nakamura et al., 2019). Unfortunately, fluorescence was always undetectable in the stable transgenic lines expressing the FP-fused LZYs. By observing the fixed and cleared roots, AtLZY3 was found to localize to the plasma membrane of columella cells. Further, the immunolocalization of eGFP-LjLZY3 in *L. japonicus* showed that LjLZY3, which controls the positive root gravitropism in *L. japonicus*, predominantly localized to the basal and apical sides of the plasma membrane in root stele cells (Chen et al., 2020).

Despite many reports consistently supporting the view that LZY homologs controlling root gravitropism are localized to the plasma membrane, other findings have indicated their nucleus localization. Whereas the full-length OsDRO1, which is encoded by a quantitative trait locus (QTL), was localized to the plasma membrane, the truncated form of OsDRO1 was localized to the plasma membrane and cytoplasm, as well as the nucleus (Uga et al., 2013). By fusing it to the rapidly maturing VENUS, Waite et al. (2020) recently found that AtLZY3 was largely localized to the nucleus of the cortical and endodermal cells of both primary and lateral root tips in the stable transgenic lines under the native conditions. Unfortunately, the fluorescence signal of this AtLZY3-VENUS still went undetected in the columella cells, implying that AtLZY3’s subcellular localization might be conditional and potentially reliant on cell type. Further investigation into the dynamic subcellular localization of LZY homologs in columella cells under native condition upon gravistimulation is now warranted, as this would provide more valuable information to address the detailed molecular mechanism of LZYs.

## Mechanism of LZYs Controlling Shoot and Root Gravitropism

Studies with mutants have revealed that LZYs control the gravitropism of shoots and roots by directing auxin flow. However, the underlying mechanism is not yet fully understood. Recently, studies of OsLZY1 and AtLZY3 have shed light on the molecular mechanism by which LZY proteins could control the plant gravitropism process.

**FIGURE 4:**
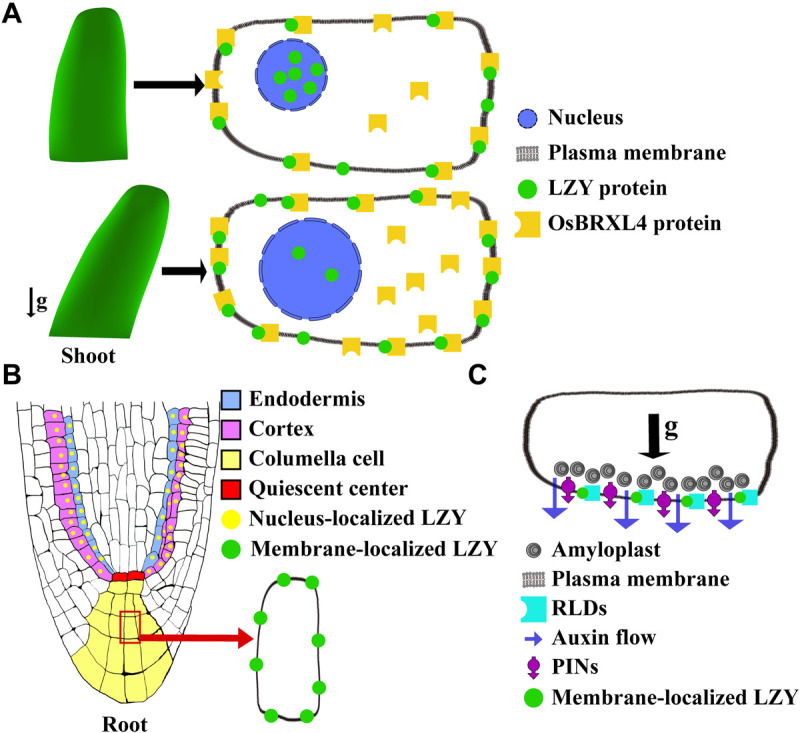
Diagrams illustrating the function of LZY proteins in regulating plant gravitropism. **(A)** In the shoot of rice, OsLZY1 interacts with OsBRXL4 to regulate shoot gravitropism. OsLZY1 is localized to both the plasma membrane and nucleus. The nuclear localization of OsLZY1 is important for its function. OsBRXL4, which bears three BRX domains and physically interacts with OsLZY1 at the plasma membrane, can regulate the proportions of membrane-localized and nucleus-localized OsLZY1 proteins. In case that OsBRXL4 is increased, the nucleus-localized OsLZY1 reduces, leading to a wider tiller angle (bottom). **(B)** The *LZY*s are expressed in the root-tip columella cells. The protein of AtLZY3 was also detected in the cortex and endodermis cells. **(C)** LZYs interact with RLDs, which bear one BRX domain, at the plasma membrane. After gravistimulation by a 90° reorientation, LZYs, RLDs, and PINs polarize along with the direction of the gravity to the bottom-side membrane of the columella cells, which further triggers PAT.

As discussed above, the C terminus is highly conserved among the LZY gene family. The studies using rice and Arabidopsis have both found that this C terminus, which harbors the EAR-Like motif, interacts with the BRX domains (Li et al., 2019; Furutani et al., 2020). In rice, OsLZY1 interacts with OsBRXL4 on the plasma membrane, where they are co-localized (Li et al., 2019). The interaction between OsLZY1 and OsBRXL4 was likely to retain OsLZY1 on the plasma-membrane, further altering the relative proportions of membrane-localized and nucleus-localized OsLZY1 proteins (Li et al., 2019; Figure 4A). Since nucleus localization is critical for OsLZY1 to exert its function, the interaction between it and OsBRXL4 profoundly influences the function of OsLZY1 and, by extension, the rice tiller angle (Li et al., 2019). When *OsBRXL4* was overexpressed, OsLZY1 was less likely to be localized to the nucleus, resulting in prostrate growth of rice tillers, which mimics the phenotype of the *oslzy1* mutant. By contrast, in the RNA interference (RNAi) lines, in which multiple *OsBRXL* homologs were targeted, OsLZY1 was more likely to be localized to the plasma membrane, resulting in more compact shoot architecture (Li et al., 2019). These results indicated that the balanced dynamic subcellular localization of OsLZY1 between the nucleus and plasma-membrane is crucial for determining its function and thus rice shoot architecture (Figure 4A).

Recently, AtLZY3 was also reported to interact with the BRX domain of Regulator of Chromosome Condensation 1 (RCC1)-like domain (RLD) proteins (Furutani et al., 2020). By imaging the roots that have been fixed and cleared, AtLZY3 was found localized to the plasma membrane of columella cells. Upon gravistimulation, AtLZY3 polarized to the new bottom side columella cells and recruited RLDs onto the plasma membrane by interacting with them via the C terminus (i.e., the domain V), thereby triggering relocalization of PIN3 and a downstream reaction (Furutani et al., 2020; Figures 4B,C). RLDs have also been called PRAF family proteins (Briggs et al., 2006). Besides the tandem repeat of the RCC1 domain at the middle and the BRX domain at the C terminus, RLDs also harbor two other conserved domains: the pleckstrin homology (PH) domain at the N terminus, and the FYVE zinc-finger domain situated between the RCC1 and BRX domains (Briggs et al., 2006; Wywial and Singh, 2010). RLDs appear to regulate auxin transport by modulating PINs’ expression and localization (Furutani et al., 2020). Thus, AtLZY3 regulates auxin transport and root gravitropism by recruiting RLDs to the plasma membrane to regulate PINs’ relocalization (Figures 4B,C). It is noteworthy that the FYVE domain is known for its function in membrane trafficking (Hu et al., 2002). Thus, it is plausible that RLDs-LZYs might represent a pathway operating to regulate PINs’ polarization by a novel type of vesicle trafficking mechanism.

As discussed above, the LZYs’ subcellular localization is a substantial component of the molecular mechanism underpinning LZYs’ activity in gravitropism. Given the current reports that LZYs may demonstrate varied subcellular localizations depending on the cell type and conditions, more investigation of the dynamic localization of AtLZY3 and other LZY homologs in both statocyte cells and other tissues will help to uncover the subcellular localization profile and mechanism of the LZYs that control root gravitropism. Nevertheless, the finding that LZYs interact with BRX domain-containing proteins to regulate auxin transport is a monumental step forward in elucidating the mechanism by which LZYs control plant gravitropism, albeit one that could be species-specific.

## Functional Divergence of the *LZY* Family

**FIGURE 5:**
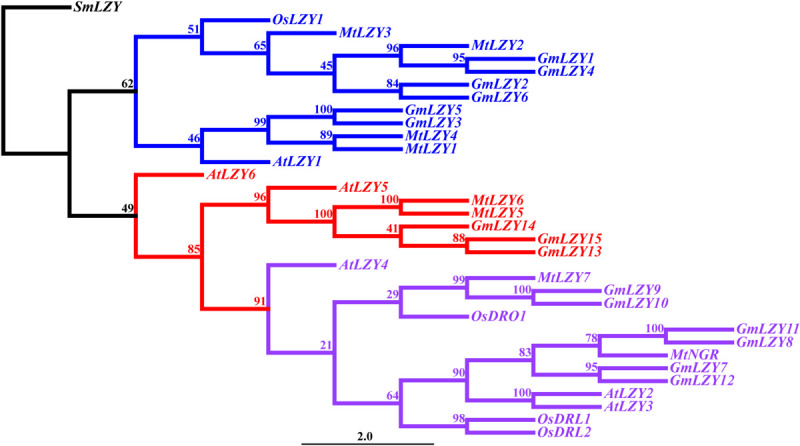
Phylogenetic analysis of LZYs among different species. The phylogenetic tree was reconstructed using the maximum-likelihood method with 1,000 bootstrap repeats. Blue represents the clade that mainly regulates shoot gravitropism. Purple represents the DRO1 clade. Red represents the clade with a currently unknown function. SmLZY served as the outgroup when constructing the tree. The numbers on the branches indicate the bootstrap values. *Sm*, *Selaginella moellendorffii*; *Os*, *Oryza sativa*; *Mt*, *Medicago truncatula*; *Gm*, *Glycine max*; *At*, *Arabidopsis thaliana*.

*LZY* genes have tended to form a small gene family in higher plants, such as Arabidopsis, rice, *M. truncatula*, and *L. japonicus* (Yoshihara et al., 2013; Nakamura et al., 2019; Figure 5 and Table 1). In Arabidopsis, there are six members in the gene family. Since the function of *AtLZY5* and *AtLZY6* in gravitropism has not been studied yet, we will not discuss them and their homologs in other species. According to mutant studies and our phylogenetic analysis (Figure 5), the *LZY*s in Arabidopsis have diverged in their functioning across varied organs. Specifically, *AtLZY1* regulates shoot and inflorescence stem gravitropism, whereas *AtLZY4* regulates root gravitropism. *AtLZY2* and *AtLZY3*, however, regulate both shoot and root gravitropism (Yoshihara et al., 2013; Ge and Chen, 2016; Taniguchi et al., 2017; Yoshihara and Spalding, 2017; Nakamura et al., 2019). The synteny analysis indicated *AtLZY2*, *AtLZY3*, and *AtLZY4* are located within the micro-syntenic blocks on chromosome 1 (Ge and Chen, 2016). It is known that the Arabidopsis genome has undergone three whole-genome duplications (WGD) events in its evolution, resulting in 60% of genes possessing paralogs in the corresponding syntenic fragments (Bowers et al., 2003; Panchy et al., 2016). The syntenic relationships of *AtLZY2*, *AtLZY3*, and *AtLZY4* suggest their three LZYs are derived from the WGD events of Arabidopsis. The divergence of *LZY*s in regulating gravitropism of different organs seems correlated with their spatial expression pattern, in that *AtLZY1* and *AtLZY4* are expressed mainly in shoots and roots, respectively, whereas *AtLZY2* and *AtLZY3* are expressed in both shoot and root. In a recent study, expression pattern divergence of gene pairs derived from WGD was observed in Arabidopsis (De Smet et al., 2017), thus explaining the divergence of the *LZY*s.

The *LZY*s in rice also displayed evidence of functional divergence. *OsLZY1* controls the tiller angle by regulating shoot gravitropism (Li et al., 2007; Yoshihara and Iino, 2007; Li et al., 2019), whereas OsDRO1, OsDRL1, and OsDRL2, which were phylogenetically clustered into the DRO1 group (Hollender and Dardick, 2015; Figure 5), regulate the root gravitropic response and hence the growth angle of both seminal and crown roots (Uga et al., 2013; Kitomi et al., 2020). The *lzy* mutants with negative gravitropic responses in their roots were also identified in the two legume species *M. truncatula* and *L. japonicus* (Ge and Chen, 2016; Chen et al., 2020). These two legume mutants resembled the *atlzy2/atlzy3/atlzy4* triple mutant of Arabidopsis (Ge and Chen, 2016; Chen et al., 2020). Notably, the causative genes of these legume mutants lie within the syntenic genome fragments, which are also syntenic to those genome blocks harboring *AtLZY2*, *AtLZY3*, *AtLZY4*, and *OsDRO1* (Ge and Chen, 2016). According to the synteny analysis, OsDRO1, OsDRL1, and OsDRL2 are paralogs, having a similar function in regulating root gravitropism in rice (Ge and Chen, 2016; Kitomi et al., 2020). It is possible that knocking-out these three paralogs may strongly enhance the gravitropic defect observed in the near-isogenic lines (Uga et al., 2013; Kitomi et al., 2020), or even completely reverse the positive gravitropism to negative gravitropism in roots, thereby resembling the legume root mutants and Arabidopsis *atlzy2/atlzy3/atlzy4* triple mutants.

Interestingly, both *OsDRO1* and *OsDRL1* are QTLs harboring natural variation among rice populations, and this contributes to the varied RSA in cultivated rice, suggesting that variation in *LZY* genes may strongly affect rooting depth and plant rooting strategies (Uga et al., 2013). As discussed previously, rooting depth is a fundamental element of the diverse RSA and it dramatically influences a crops’ performance. Variation in *LZY*s can lead to varied gravitropic responses of roots, which further changes the GSA of the root tip, resulting in varied rooting depth. Thus, manipulating the activity of LZYs offers a very promising solution to engineering the plant RSA in crops, trees, and other economically important plants.

## *LZYs* Broadly Exist in the Plant Kingdom

Since the identification of *LZY* genes in several angiosperm plants, including maize, Arabidopsis, *M. truncatula*, *L. japonicus*, and *Prunus domestica* (Dong et al., 2013; Yoshihara et al., 2013; Howard et al., 2014; Ge and Chen, 2016; Taniguchi et al., 2017; Yoshihara and Spalding, 2017; Chen et al., 2020), similarity-based sequences searches have found the presence of LZY homologs broadly in plant genomes, including the primitive plant *Physcomitrella patens* (moss), *Selaginella moellendorffi* (fern), and gymnosperm plant *Picea sitchensis* (spruce) (Dardick et al., 2013; Yoshihara et al., 2013). As discussed above, the overall sequence similarity of these LZYs from various species is low, whereas the identity of the five domains is quite high. These conserved domains have been shown to be critical to the functioning of the proteins (Yoshihara and Spalding, 2020), thus, it is possible that LZYs from different species may share functional conservation to a certain extent. Additionally, all *LZY* genes from moss to higher plant preserved a very similar intron-exon pattern, particularly the first six base pairs of the coding sequence immediately after the five prime untranslated regions (5′UTR) of the first exon (Dardick et al., 2013). The comparative genomic analysis also found that *LZY* family members are located within the syntenic genome fragments from monocot to dicots (Ge and Chen, 2016). All these findings concur, by extensively demonstrating the high conservation of LZYs, whose corollary is that LZYs share a common evolutionary origin.

## LZYs Belong to Igt Gene Family

While mutations of *LZY1* genes leads to impaired shoot gravitropism, wider tiller angles, or a prostrate growth habit in diverse species, mutation of *Tiller Angle Controller 1* (*TAC1*) confers a narrower tiller angle in rice, lateral axillary branches in Arabidopsis, and pillar shoot architecture in trees, the opposite phenotypic traits of *lzy1* mutants (Yu et al., 2007; Dardick et al., 2013; Hollender et al., 2018). LZY proteins and TAC1 share similar sequence structures and conserved domains, except that TAC1 lacks the critical domain V that contains the EAR-like motif. Phylogenetic analysis suggested that LZYs and TAC1 constituted two clades of the IGT gene family, which is named after the conserved unique motif (GφL(A/T)IGT) located within the second domain of LZY and the TAC1 gene family (Dardick et al., 2013; Hollender and Dardick, 2015).

Given the similar sequence pattern but opposite consequences arising from mutation of *LZY1* and *TAC1*, the relationship between *LZY1* and *TAC1* and the hidden mechanism responsible for the opposite effects is very interesting. Might there be a direct connection or genetic/physical interaction between these two proteins? A recent work has revealed that *LZY1* and *TAC1* do show a similar expression pattern in shoot tissues in Arabidopsis (Hollender et al., 2020). The double mutant analysis indicated that *lzy1* was epistatic to *tac1* with respect to the shoot’s branch angles and orientation (Hollender et al., 2020). Unlike LZY1, which controls shoot architecture by modulating gravitropism, the shoot of *tac1* normally responds to gravitation pull (Hollender et al., 2020), implying that the regulatory and interaction mechanism underlying LZY1 and TAC1 is rather complex and goes beyond a pure gravitropic regulation.

While LZY genes widely exist in terrestrial plants, including primitive taxa, TAC1 was not found in the latter, but it is present in the vascular plants with lateral axillary organs. Thus, it is plausible that TAC1 is evolved from LZY genes as a truncated LZY (Hollender and Dardick, 2015). The emergence of TAC1 in plant genomes appears to be associated with the origination of lateral shoots in plants evolution (Dardick et al., 2013; Hollender and Dardick, 2015). The counterbalancing effects between LZY1 and TAC1 may have provided a delicate mechanism for attaining the diverse shoot architecture seen among higher plants.

## Conclusion and Perspectives

Due to their sessile nature, plants cannot move away from the microenvironment where they are established. Yet, plants can sensitively detect various environmental signals, such as light, temperature, gravitation pull, and water availability. Beyond that, plants have evolved complex mechanisms, by which they can utilize environmental signals as guides to adjust their growth, physiological status, and even behavior, in a plastic manner, thereby achieving a better fit to the current environment in which they live. In this context, gravity can be viewed as an important plant growth regulator, since plants’ fitness depends on orienting their tissues properly along its field.

The process of gravitropism, which is triggered by gravitational pull and results in the bending of plant organs, offers plants better adaptation by guiding their roots to grow deep into the soil to uptake water and nutrients, and steering shoots to grow away above the ground to capture incident sun radiation needed for photosynthesis and reproduction. This process, requiring the redistribution of auxin under the guide of gravity, was an evolutionary milestone, in going from ancestral aquatic life, which existed only in the buoyancy environment, to land plants able to sense and respond to gravitational pull properly. LZY genes are among the essential components of the plant gravitropism chain, since mutations of LZYs result in the failure of proper gravitropic responses, in both shoots and roots. LZYs have been widely found in land plants, including the primitive lineages, but not in the aquatic algal genome (Dardick et al., 2013; Yoshihara et al., 2013); this suggests that the emergence of LZY genes may have contributed to the establishment of gravitropism in land plants, thus conferring to them a better fitness within the gravitational field of the Earth.

Gravitropism is a fascinating subject and a focus of plant science study for centuries. However, the molecular architecture of this highly complex form of tropism is largely murky, particularly concerning the details of signal perception through to asymmetric auxin redistribution (Nakamura et al., 2019). Recent research from multiple species has consistently demonstrated that LZYs’ mutation alters the auxin transport in the shoot, and reversed auxin asymmetry in the root tip. By identifying an important player in the early events of plant gravitropism process, this body of work has also opened a new doorway to investigate the molecular mechanism triggering the relocation of auxin carriers and corresponding asymmetrical auxin flux.

The subcellular localization of LZYs varies under different experimental conditions, which may imply a naturally dynamic and conditional localization of these proteins, potentially involving the movement of LZYs among certain organelles. The recent finding that AtLZY3 is expressed in endodermal and cortical cells in root tips is intriguing, as it implies an unknown mystery behind LZYs. Subcellular localization and tissue-specific expression pattern are the fundamentals to understand the function of a gene. Thus, future efforts to further address the dynamic subcellular localization and tissue-specific expression pattern of LZYs will undoubtedly strengthen the current picture of LZYs as the key regulators of gravitropism. That both BRXs and RLDs can interact with LZYs represents a remarkable breakthrough in unraveling how LZYs mechanistically control gravitropism, and also represents future directions to explore in further resolving the molecular architecture of plant gravitropism, which possibly involves a new type of vesicle trafficking that regulates the movement of LZYs within cells.

In conclusion, the identification and studies of LZY genes have provided fresh and exciting insight into the mechanism of plant gravitropism; future studies into the genetic and molecular details of the LZY-dependent signaling pathway upon gravistimulation will establish the connection between LZYs and downstream reaction, significantly advancing our understanding of the most mysterious part of plant gravitropism.

## Author Contributions

ZJ and LG wrote the manuscript. HD, SC, and WH revised the manuscript. All authors read and approved the manuscript.

## Conflict of Interest

The authors declare that the research was conducted in the absence of any commercial or financial relationships that could be construed as a potential conflict of interest.

## Abbreviations

RSA: root system architecture
GSA: gravitropic set-point angles
PAT: polar auxin transport
SOR: soil-surface roots
BiFC: bimolecular fluorescence complementation
NLS: nuclear localization signal
GFP: green fluorescence protein
MTFs: membrane-bound transcription factors
QTL: quantitative trait locus
RNAi: RNA interference
WGD: whole-genome duplications
5′UTR: five prime untranslated regions.
